# Transforming infectious disease control through social innovation, community engagement and intersectional gender research

**DOI:** 10.1136/bmjgh-2026-023522

**Published:** 2026-03-19

**Authors:** Meredith Labarda, Uche Amazigo, Sushil Chandra Baral, Beatrice Halpaap, Lenore Manderson, Mariam Otmani Del Barrio

**Affiliations:** 1 Medicine, University of the Philippines Manila School of Health Sciences, Tacloban, Leyte, Philippines; 2 Program on Social Innovation and Entrepreneurship in Health (SIHI Philippines Hub), Institute of Clinical Epidemiology, University of the Philippines Manila, National Institutes of Health, Manila, NCR, Philippines; 3 Pan-African Community Initiative on Education and Health, Enugu, Nigeria; 4 Research, HERD International, Kathmandu, Nepal; 5 World Health Organization Special Programme for Research and Training in Tropical Diseases, Geneva, Switzerland; 6 School of Public Health, University of the Witwatersrand, Parktown, Johannesburg, Gauteng, South Africa; 7 Institute at Brown for Environment and Society, Brown University, Providence, South Africa; 8 UNICEF/UNDP/World Bank/WHO Special Programme for Research and Training in Tropical Diseases (TDR), World Health Organization, Geneva, Switzerland; 9 TDR, World Health Organization, Geneva, Switzerland

**Keywords:** Public Health, Tuberculosis, Health policy

## Abstract

Community engagement and approaches that aim to change unequal power relations are essential for inclusive, relevant and sustainable health interventions. A people-centred approach to research and programme implementation can amplify the voices of disadvantaged and often forgotten people and move towards genuine partnership with the communities, ensuring that research and action meaningfully reflect the priorities and realities of those most affected.

**Summary box**

- Community engagement and an intersectional gender approach in infectious disease research and programmes promote inclusive and equitable access to healthcare and redress unequal power relations.
- Locally owned and context-responsive approaches improve the relevance, uptake and effectiveness of both research and interventions in settings of poverty.
- Strengthening local capacity enables community-driven social innovation in health research and programmes and supports more equitable and resilient health outcomes.

## Introduction

 Global power imbalances still shape how infectious disease research and health programmes are designed, with institutions in high-income countries often setting the agenda while the most-affected communities have limited influence. This approach can limit the effectiveness of interventions, particularly in light of gender norms, structural inequities and historical injustices that may influence health outcomes. Moving towards genuinely people-centred health means recognising communities as equal partners. Ethically responsive, gender-transformative and community-based stakeholder engagement is essential to address the unequal power dynamics that impact healthcare delivery and access in Low and Middle Income Countries (LMICs).^1^

Using examples from Africa, the Philippines and Nepal, we show how community engagement is essential for ensuring flexibility and responsiveness to local contexts when conducting implementation research and delivering infectious disease interventions in settings of poverty. In addition, an intersectional gender approach can help researchers and practitioners move away from a ‘one-size-fits-all’ or ‘blueprint’ approach in both the design and execution of research studies and health interventions. This ensures that research is precise and adaptable to existing health services or able to develop new interventions. Health interventions that are responsive to local contexts are more likely to be accepted and effective on the ground. Additionally, their results are more relevant for policy and are more effective in guiding interventions for specific populations.^2^

## Community engagement in health programmes and infectious disease research

Community engagement is fundamental to both health programmes and infectious disease research because it helps ensure that decisions are shaped not only by scientific evidence but also by community priorities and lived realities. Community engagement involves the meaningful participation of community members with other stakeholders in the design and implementation of interventions, as well as modifying existing practices or introducing new approaches.^2 3^ For example, Uganda, Ghana, Nigeria and Cameroon have had success in using community engagement strategies to improve access to medicines for neglected tropical diseases, as illustrated by the Community Directed Treatment with Ivermectin (CDTI) and Community Self-Monitoring (CSM) models.^4^,^7^ Multicountry studies on CDTI showed that coverage rates were higher in programmes designed by communities than those using routine health service delivery methods, with adequate treatment coverage of at least 80% of the total affected population.^8 9^ This increase in coverage also translated to an increase in the number of people treated from 1.5 million in 1997 to 100.79 million in 2013.^10^

Building on these examples, the Social Innovation in Health Initiative piloted a Community Engagement Self-Monitoring (CESM) strategy in the Philippines,^11^ with the support of TDR, the Special Programme for Research and Training in Tropical Diseases.^12^ In 2021, CESM was piloted for two community-managed social innovations, one at an urban tuberculosis clinic^13^ and the second through a rural health monitoring programme.^14^ The CESM strategy involves communities in the selection of local monitors and empowers them to develop, design and implement their own self-monitoring tools. Monitors are selected based on community trust, prior engagement and local knowledge. Feedback mechanisms and peer learning spaces are integral, creating ‘safe spaces’ for discussion, collective learning and encouragement.^15^ Both the African CDTI strategy and the Philippine CESM approach emphasised strong community decision-making, trust in local volunteers and leadership support. In both contexts, regular training enhanced competencies in data use and encouraged meaningful participation in local governance.^12 16^

Shifting towards community-driven solutions in health also implies fully integrating community engagement in infectious disease research. This calls for ethical, respectful and fit-for-purpose involvement of community members within the research process, from involvement during the identification of the study to defining its purpose and design and its implementation, interpretation and use of results stages. Participatory action research (PAR) is one approach which offers this. PAR encourages community and health systems actors to identify local problems and create solutions that can promote social change. Co-learning between researchers and communities encourages collaborative problem identification, action and reflection.^16^ PAR prioritises those who are less powerful and encourages researchers and practitioners to challenge their own position and power. Breaking down power relations is essential to developing person-centred health systems and enabling sustained health development and social change.^17 18^

## Why community engagement needs an intersectional gender approach

Power dynamics and inequities can influence different aspects of the research cycle, from participant selection to data collection and analysis.^19^ Applying an intersectional gender lens in health research contributes to a shift towards balancing power dynamics, facilitating and enabling the co-creation of community-driven research. This helps reduce potential harm stemming from researchers’ own positionalities and supports a community-driven understanding of diverse health concerns, including how people experience difference, discrimination or privilege.^20^

One way to systematically explore these intersecting forms of inequality is through intersectional gender analysis, which provides a framework for identifying how different social factors, within connected systems and structures of power, shape health experiences under different forms of privilege and oppression. Intersectional gender analysis examines how gender power relations intersect with other social factors to affect people’s lives and create differences in needs and experiences. It also analyses how policies, services and programmes can help to address these differences. For example, integrating such analyses into routine health programmes can offer insights into how gender, age, ethnicity, socioeconomic status and occupation influence disease conditions, access to care and health outcomes in a given context.^20 21^ It also responds to the needs of diverse population subgroups, recognising that different women and men do not necessarily face the same health challenges and needs.

For example, a study in central Uganda funded by TDR used intersectional gender analysis in tuberculosis care and illustrated how men and women experienced health service delivery differently. While women still waited longer to be diagnosed than men, those over 40 years old, specifically in rural areas, faced the greatest risk of tuberculosis infection but also a greater delay in diagnosis.^4^ Another example also supported by TDR can be found in a study conducted in a Ugandan district, which used intersectional gender analysis in analysing community and health system level interventions to curb the infection of schistosomiasis.^22^ This research showed that gender intersects with education and income levels so that vulnerability to schistosomiasis and access to treatment occurred within a complex web of gender relations, poverty, limited economic opportunities and insufficient health service delivery.

## The case of lymphatic filariasis and tuberculosis in Nepal

Lymphatic filariasis and tuberculosis remain major challenges in Nepal^23^ despite government efforts such as repeated mass drug administration campaigns and various community health interventions. Nepal is a country with wide sociocultural diversity, with 125 ethnic groups and 123 languages spoken. Furthermore, persistent inequities that are derived from deeply rooted gender norms, economic disparities and geographical barriers influence the changing nature of the disease.

Using an intersectional gender analysis, TDR-funded research showed how gender and ethnicity intersect and shape vulnerability, particularly in Madhesi and Tharu communities.^24^ Men missed drug campaigns due to outdoor occupations and frequent travel. Women faced barriers to accessing services due to their socially assigned caregiving roles and restricted mobility. Older people disregarded the importance of drug campaigns in disease control.^23 24^

As a result of the findings, Nepal has revised community-based interventions and incorporated gender-specific strategies^23 25^ to include distributing drugs to underserved children in schools, as well as shifting drug distribution responsibilities from female community health volunteers to health workers in urban areas to address compliance and trust issues.^24^ Early results show that these campaigns have significantly reduced the at-risk population from 25 million to 9.8 million in the fiscal year 2022–2023, and most districts now have over 80% epidemiological coverage.^24^

In the case of TB programmes, research revealed significant differences in health outcomes by age, sex and social factors like caste and homelessness.^24 26^ These complexities could only be addressed through a deeper grasp of the programme context and the ways challenges intersect within communities. Strategies target high-risk populations, including poor urban people, slum dwellers, factory workers and people on daily wages. Advocacy and communication campaigns aim to combat stigma and promote treatment concordance among vulnerable groups.^27^ Training of community health workers, instrumental in connecting communities and health services, especially in underserved areas,^28^ now includes equity and social inclusion, with a gender-transformative approach.^29^

## Conclusions

The case of Nepal’s research findings and its consequent use to inform policy and practice highlights the benefits of integrating intersectional gender analysis into infectious disease control. To make this more effective, it is vital to (a) ensure local ownership of both the research process and the design, delivery and implementation of interventions; (b) continue investing in collecting and analysing disaggregated data with an intersectional gender lens to capture how health needs and outcomes differ across sexes, gender identities, ages, socioeconomic status and other social factors; (c) build the capacity of local health systems, frontline health workers and community groups to interpret evidence and adapt programmes accordingly and (d) engage local governments by involving them early and aligning research findings with local policy and planning.

A people-centred approach to infectious disease research and programme implementation requires more than technical solutions. It demands genuine partnership with the communities most affected and an honest reckoning with the power dynamics that shape global health. By grounding research and programmes in community priorities and shared decision-making, this approach strengthens trust, relevance and long-term impact. It reflects a community-driven understanding of diverse health concerns: how and why individuals experience difference, discrimination or privilege and how gender inequities manifest, intersect with and are influenced by other drivers of inequality.^20^

## Data Availability

Data are available upon request.
